# Graphene-enabled and directed nanomaterial placement from solution for large-scale device integration

**DOI:** 10.1038/s41467-018-06604-4

**Published:** 2018-10-05

**Authors:** Michael Engel, Damon B. Farmer, Jaione Tirapu Azpiroz, Jung-Woo T. Seo, Joohoon Kang, Phaedon Avouris, Mark C. Hersam, Ralph Krupke, Mathias Steiner

**Affiliations:** 1 IBM Research, Rio de Janeiro, RJ 22290-240 Brazil; 2 IBM Research, Yorktown Heights, NY 10598 USA; 3 Department of Materials Science and Engineering and Department of Chemistry, Northwestern University, Evanston, IL 60208 USA; 4 Institute of Nanotechnology, Karlsruhe Institute of Technology, 76021 Karlsruhe, Germany; 5 DFG Center for Functional Nanostructures (CFN), 76028 Karlsruhe, Germany; 6 Institut für Materialwissenschaft, Technische Universität Darmstadt, 64287 Darmstadt, Germany

## Abstract

Directed placement of solution-based nanomaterials at predefined locations with nanoscale precision limits bottom-up integration in semiconductor process technology. We report a method for electric-field-assisted placement of nanomaterials from solution by means of large-scale graphene layers featuring nanoscale deposition sites. The structured graphene layers are prepared via either transfer or synthesis on standard substrates, and then are removed once nanomaterial deposition is completed, yielding material assemblies with nanoscale resolution that cover surface areas >1 mm^2^. In order to demonstrate the broad applicability, we have assembled representative zero-dimensional, one-dimensional, and two-dimensional semiconductors at predefined substrate locations and integrated them into nanoelectronic devices. Ultimately, this method opens a route to bottom-up integration of nanomaterials for industry-scale applications.

## Abstract (web summary)

The placement of nanomaterials at predefined locations is a key requirement for their integration in nanoelectronic devices. Here, the authors devise a method allowing placement of solution-based nanomaterials by using structured graphene layers as deposition sites with the aid of an electric field.

## Introduction

Controlled placement of nanomaterials at predefined locations with sub-micron precision remains among the most challenging problems that inhibit their large-scale integration in the field of semiconductor process technology. Methods based on surface functionalization^1^ have a drawback where undesired chemical modifications can occur and deteriorate the deposited material. The application of electric-field-assisted placement techniques^2^ eliminates the element of chemical treatment; however, it requires an incorporation of conductive placement electrodes that limit the performance, scaling, and density of integrated electronic devices^3^. Here, we report a method for electric-field-assisted placement of solution-processed nanomaterials by using large-scale graphene layers featuring nanoscale deposition sites. The structured graphene layers are prepared via either transfer or synthesis on standard substrates, and then are removed once nanomaterial deposition is completed, yielding material assemblies with nanoscale resolution that cover surface areas >1 mm^2^. In order to demonstrate the broad applicability, we have assembled representative zero-dimensional, one-dimensional, and two-dimensional semiconductors at predefined substrate locations and integrated them into nanoelectronic devices. This graphene-based placement technique affords nanoscale resolution at wafer scale, and could enable mass manufacturing of nanoelectronics and optoelectronics involving a wide range of nanomaterials prepared via solution-based approaches.

Bottom-up, large-scale manufacturing of integrated electronics requires application of substrate patterning, chemical surface functionalization, and/or Langmuir-Blodgett-type techniques for depositing solution-based, semiconducting materials at the substrate’s surface^4^. Chemistry-based techniques can be applied to deposit nanomaterials at wafer scale^5^; however, they offer limited control over the material’s location, orientation, and density. In addition, the use of aggressive chemicals may lead to inferior device performance once the integration process is completed.

As an alternative method, the application of electric-field-assisted deposition requires the presence of conductive electrodes for generating an attractive force that drags nanomaterials to predefined locations where material deposition is desired^6^. The method was applied successfully to integrate low-dimensional semiconductors in exploratory electronic and optoelectronic devices; for example, zero-dimensional quantum dots (0D)^7^, one-dimensional nanowires and nanotubes (1D)^8,9^, and two-dimensional graphene^10,11^. However, conductive electrodes are difficult to remove after deposition, typically require harsh chemistry or ion/atom bombardment, limiting electronic device performance and integration density^3^. Graphene electrodes, on the other hand, can be removed quite easily by a mild oxygen plasma without adversely affecting the substrate or nanomaterial. In order to harvest the nanoscale placement potential of the dielectrophoresis (DEP) method at large scale and, at the same time, to avoid the use of unwanted conductive electrodes in the placement process, another solution is needed. Graphene, an excellent candidate material for electric-field-assisted material deposition, offers a potential solution to this, as it can be removed almost residue-free in a standard etching procedure^12^. In addition, this material possesses a combination of properties critical to this placement technique, including AC operation capability^13,14^, wafer-scale growth^13,14^ or transfer onto a wide range of standard substrates^15^, and nanometer resolution patterning^16^.

## Results

### Conception of graphene-enabled nanomaterial placement

**Fig. 1:**
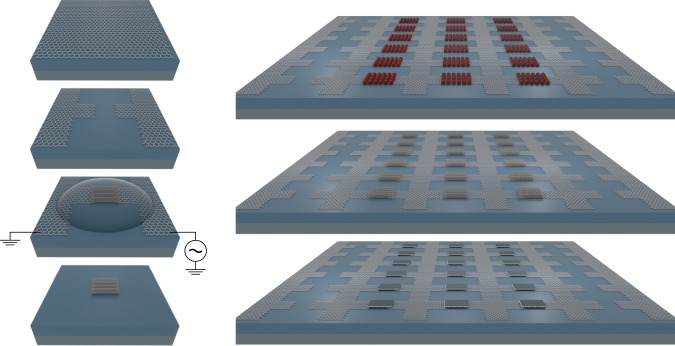
Electric-field-assisted placement of nanomaterial from solutions with patterned graphene. Artistic rendering of electric-field-assisted placement of nanoscale materials between pairs of opposing graphene electrodes structured into a large graphene layer located on top of a solid substrate. The sketches on the left-hand side visualize the key steps of the method: structuring a large-scale graphene layer to form local electrodes, applying a AC voltage for facilitating the field-assisted placement of nanomaterials from solution (shown are carbon nanotubes), and removing the graphene structure once the placement is complete. In the visualization on the right-hand side, quantum dots (0D), single-walled carbon nanotubes (1D), and layers of molybdenum disulfide (2D) are shown as representative nanomaterials that can be assembled at large scale based on the graphene-based, electric-field-assisted placement method

In Fig. 1, the principle of the electric-field-assisted placement method using graphene electrodes is visualized for three representative nanomaterials. A large graphene layer is patterned to form sets of opposing electrodes at predefined positions on top of a solid substrate. The application of an alternating voltage between the graphene layers generates local electric fields at predefined substrate positions. If nanomaterial dispersions are placed on top of the substrate, a dielectric force attracts the nanomaterials towards the positions defined by the graphene electrodes where the nanomaterials settle at the substrate surface. Finally, the graphene layers are removed leaving behind the nanomaterials assembled at the predefined substrate positions. While large-scale assembly using metal deposition electrodes has achieved high carbon nanotube (CNT) density^8^, it does not offer the full flexibility with regards to device contact and circuit interconnect design. In addition to its excellent properties as an AC conductor^13,14^, graphene offers optimal geometrical placement conditions for nanomaterials in comparison with the much thicker metallic electrodes typically used in electric-field-assisted deposition methods^8^.

**Fig. 2:**
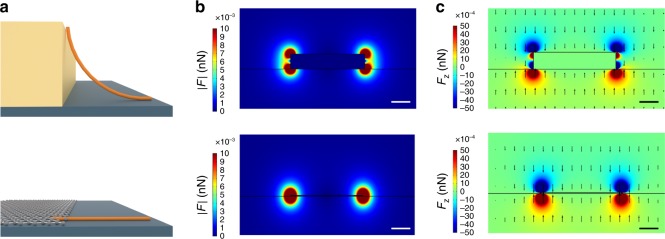
Geometrical and electrostatic conditions in graphene-based placement of nanomaterials. **a** Cross-section of a standard metal electrode used for electric-field-assisted assembly of nanomaterials and a graphene layer, having a thickness of 1.5 nm in our experiments, which is used for the same purpose. Both electrodes are located on top of a solid substrate. As a reference placement example, a carbon nanotube is shown that bridges contact and substrate. Cross-section of the **b** dielectrophoresis force distribution and **c** dielectrophoresis force *z*-component exerted on a carbon nanotube in aqueous solution which is generated by a dc voltage applied to the standard metal electrode (top) and the graphene layer (bottom), respectively. Scale bar: 50 nm

In Fig. 2, we visualize the geometrical cross-sections and the dielectric force distributions at the electrode’s contact edge (see also Supplementary Figure 1). As an ideal 2D nanomaterial with atomic thickness, graphene enables reduction of the thickness of conventional deposition electrodes by two orders of magnitude, from several tens of nanometers (metal electrodes) to less than one nanometer. The electric field distribution is strongly localized at the substrate surface, providing ideal deposition conditions in close vicinity of the graphene layer edge. In comparison with a standard metal electrode, the deposited nanomaterial exhibits significantly lower bending at the contact edge, which reduces the likelihood of device breakdown^17,18^.

### Graphene-enabled and directed placement of CNTs

**Fig. 3:**
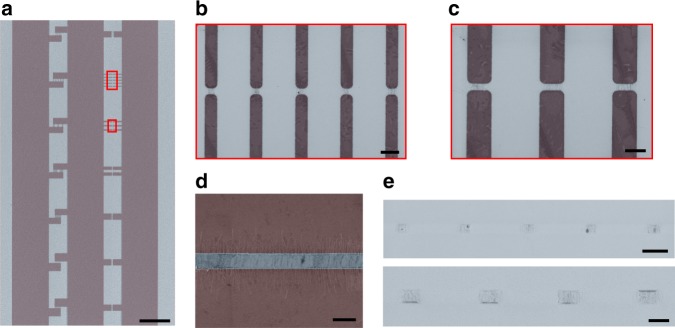
Pitch and orientation in graphene-based, electric-field-assisted nanomaterial placement. **a** Scanning electron microscope (SEM) false color image of a graphene layer on a SiC substrate featuring test structures of varying size and orientation for AC field-assisted assembly of carbon nanotubes from solution. The red lines frame the areas that are shown in **b**, **c**. Scale bar: 50 µm. **b**, **c** Magnified SEM false color images taken at the positions highlighted by dashed frames showing a representative graphene deposition structures after carbon nanotube placement is completed. Scale bars: 2 µm. **d** SEM false color image of an extended graphene electrode pair after placement is completed, exhibiting position and orientation of carbon nanotubes with respect to the gap. Scale bar: 1 µm. **e** SEM false color images of carbon nanotube assemblies after removal of the graphene layer leaving only CNTs and some residues. The upper panel corresponds to the area imaged in **b**. Scale bar: 2 µm. Deposition conditions are *V*_DEP_ = 3 V, *f* = 1 MHz, *t* = 5 min

An example of a structured graphene layer for electric-field-assisted nanomaterial deposition is shown in Fig. 3. A large-scale graphene layer (mm-scale) features smaller structures (μm to nm scale) designed for investigating deposition quality as a function of pitch. The microscopy images demonstrate the design flexibility for the graphene-based deposition electrodes, and details regarding their fabrication are provided in the Methods section. In our experiments, we have covered the entire graphene layer system uniformly with aqueous solution containing spatially isolated, semiconducting CNTs as a reference material for nanomaterial deposition^19^. Upon application of the deposition voltage *V*_DEP_, as visualized in Fig. 1, CNT placement occurs simultaneously across the solution-covered substrate surface at locations framed by opposing graphene electrode pairs. Generally, the material density can be adjusted by tuning *V*_DEP_ and the nanoparticle concentration in solution. For a given set of parameters, we obtain a constant and uniform placement density, largely independent of the graphene electrode size. In the examples shown in Fig. 3, a rather constant nanotube density of about 15 μm^−1^ is maintained (see also Supplementary Figures 2, 3). Importantly, no CNTs are found at positions outside the predesigned electrode areas. We therefore conclude that size and position of deposition sites are mainly determined by the graphene pattern. For analyzing the density and orientation of nanotubes within the deposition area, we have designed laterally extended electrode pairs like the one shown in Fig. 3d. As expected from the electric field distribution and the nanotube geometry (see Fig. 2), the CNTs align symmetrically across the gap with a density of about 15 μm^−1^ at an average angle of (90±10)°. We note that increasing either deposition voltage or concentration of nanoparticles in solution increases the material density in the placement, to values above 50 μm^−1^. Overall, this placement of individual nanotubes and nanotube bundles in aligned arrays with a homogeneous density is highly suitable for scalable manufacturing of high-performance electronic devices.

### Graphene-enabled placement of quantum dots and nanosheets

**Fig. 4:**
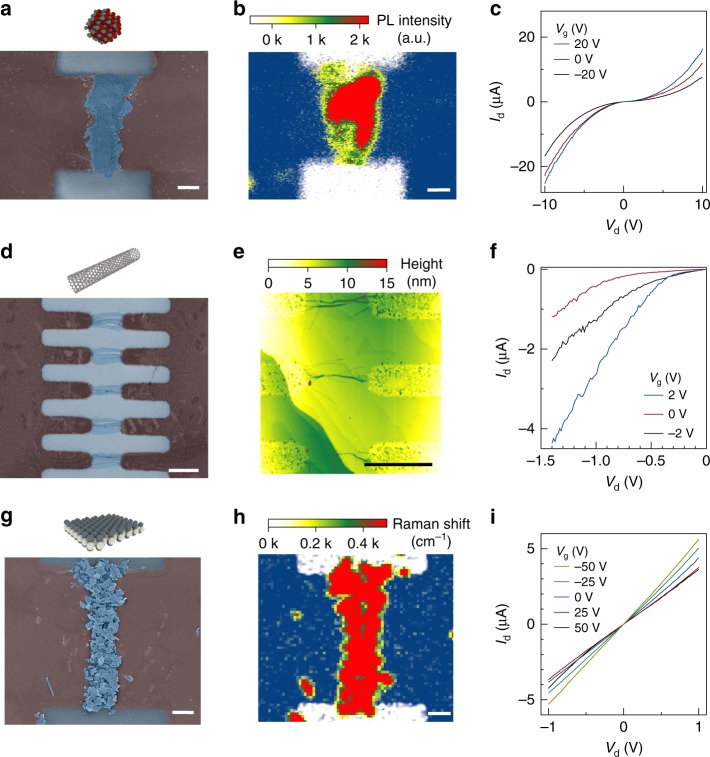
Spatially resolved deposition, characterization and device integration of 0D, 1D, and 2D semiconductors. **a** Scanning electron microscope (SEM) false color image of a CdSeS/ZnS quantum dot assembly. Deposition conditions are *V*_DEP_ = 10 V, *f* = 1 MHz, *t* = 10 min. Scale bar: 1 µm. **b** Raman 2D intensity false color image (white: 20 arb. units, blue: 100 arb. units) indicating the graphene areas, overlaid by a photoluminescence intensity false color image spectrally integrated at (580 ± 10) nm indicating the position of quantum dots. The images are taken at the same area as in **a**. **c** Electrical transport characteristic of a quantum dot thin film device. Scale bar: 1 µm. **d** SEM false color image of carbon nanotube assembly. Deposition conditions are *V*_DEP_ = 3 V, *f* = 1 MHz, *t* = 5 min. Scale bar: 1 µm. **e** Atomic force microscope image of the carbon nanotube assembly imaged in **d**. Scale bar: 1 µm. **f** Electrical transport characteristic of a carbon nanotube thin film device. **g** SEM false color image of few-layer molybdenum disulfide assembly. Deposition conditions are *V*_DEP_ = 5 V, *f* = 1 MHz, *t* = 5 min. Scale bar: 1 µm. **h** Raman 2D intensity false color image (white: 20 arb. units, blue: 100 arb. units) indicating graphene areas, overlaid by a Raman intensity false color image spectrally integrated at (385 ± 10) cm^−1^ indicating the position of few-layer molybdenum disulfide. The images are taken at the same area as in **g**. Scale bar: 1 µm. **i** Electrical transport characteristic of a molybdenum disulfide thin film device

In order to investigate the broader applicability of the method, we show in Fig. 4 the material placements for three representative solution-processed, low-dimensional (0D, 1D, and 2D) semiconductors, namely CdSeS/ZnS alloyed quantum dots, semiconducting CNTs, and few-layer molybdenum disulfide (MoS_2_), respectively. The material is identified by in situ spectroscopic analysis and characterized by atomic force microscopy to confirm the chemical identity and surface coverage (see also Supplementary Figures 4–6). The results demonstrate that the method allows effective deposition of solution-based semiconducting nanomaterials, regardless of their specific material properties. In order to determine the electrical transport properties of the nanomaterial deposits, we have manufactured three-terminal field-effect devices (see Methods section). By applying appropriate bias voltages to the devices, we obtain the expected semiconducting transport characteristics for all reference materials used in this study. The results suggest that the deposition method can, in principle, be used to manufacture electronic devices from a broad range of solution-based nanomaterials.

### Large-scale integration study

**Fig. 5:**
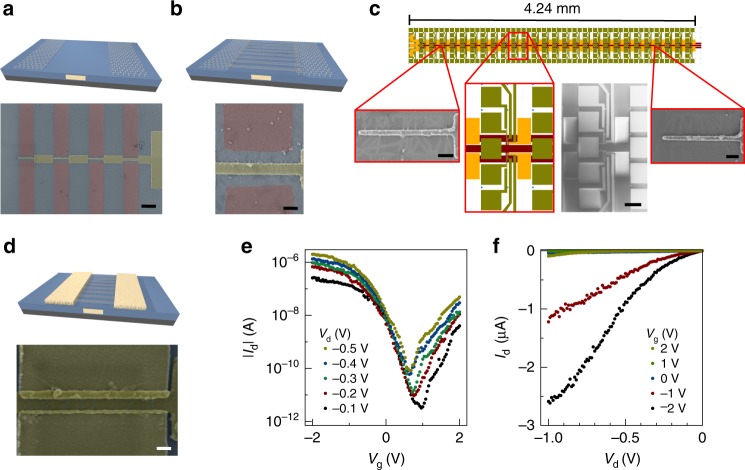
Large-scale device integration and scaling with graphene-based, electric-field-assisted nanomaterial placement. **a** Artistic rendering of a Si/SiO_2_ substrate with embedded metal electrode and a patterned graphene layer for field-assisted carbon nanotube placement. The scanning electron microscope (SEM) false color image reveals how the graphene layer (red) is patterned to form gaps at the position of the embedded metal electrode (yellow) for facilitating the nanotube placement. Scale bar: 1 µm. **b** Artistic rendering and SEM false color image of the carbon nanotube placement across the embedded metal electrode (yellow), at the substrate surface, before the graphene electrodes are removed (red). Deposition conditions are *V*_DEP_ = 3 V, *f* = 1 MHz, *t* = 5 min. Scale bar: 200 nm. **c** Layout of large-scale integration based on the device architecture in **a**–**f**. The insets display SEM images of individual devices taken at representative locations of a mm-scale assembly structure, as well as an SEM image of a larger set of individually addressable devices. Scale bars (from left to right): 200 nm, 50 µm, and 200 nm. **d** Artistic rendering and SEM false color image (top view) showing final carbon nanotube array transistor, after removal of graphene layer and manufacturing of metal contacts. Scale bar: 100 nm. **e** Electrical transfer characteristics of a carbon nanotube array transistor. **f** Electrical output characteristics of a carbon nanotube array transistor

For investigating the integration and scaling potential of the placement method, we have applied the graphene-based material deposition to an integrated SiO_2_/Si gate stack customized for three-terminal field-effect transistor integration (see Fig. 5). The gate stack contains a regular array of embedded, metallic electrodes that enables downscaling of transistor dimensions while maintaining gate coupling and planarity while considering the particular electrostatics in the design to mitigate undesired fringing fields^20^. In the present example, the minimum gate electrode length is 40 nm. For the mass production of transistors in parallel, we have patterned the graphene layers to match the position of the embedded electrodes in the substrate (see Fig. 5). In this manner, we are able to place the nanomaterials with a spatial resolution below 100 nm over areas with lateral lengths >5 mm. Electrical transfer and output characteristics of a transistor examined in a random sample from this series exhibit current on/off ratios of 10^5^ attesting to the absence of metallic CNTs in the device. While further research is needed to quantify the device yields by means of automated electrical transport analysis, we note that based on a visual inspection the deposition yields achieved in the current architecture are close to unity (see also Supplementary Figures 7, 8).

## Discussion

We note that large-scale integration of high-performance electronic circuits made from nanotube solutions, as recently demonstrated in ref. ^21^, is feasible based on the method reported here. While experiments in this work are performed at the scale of wafer dies, implementation at full wafer scale could soon be demonstrated, considering the feasibility of wafer-scale dielectrophoretic assembly with standard metal electrodes^22^. In addition, our approach enables the design of integrated circuits such as integrated photodetector or light-emitting diodes circuit where certain device functionalities are carried out by different nanomaterials. In that regard, we note that successive deposition steps are feasible with the method discussed here, that is, once the materials are deposited, they remain adhered to the substrate surface and tolerate multiple subsequent deposition and rinsing steps. Another important direction of future research is the demonstration of the graphene-based placement method on a single particle level. Since dielectrophoresis with metal electrodes has already achieved individual nanotube and nanowire precision^8,9^, an extension of our method could enable large-scale integration of exploratory quantum electronics and optoelectronics devices^23^, as well as applications in biotechnology^24^.

In summary, we have reported a method for electric-field-assisted placement of solution-processed nanomaterials by using patterned graphene layers that can be removed after the deposition of nanomaterials is completed. This method is compatible with conventional semiconductor processing and can be applied to a broad class of nanomaterials and substrates used in industrial-scale production. It is amenable to further device scaling and complex integration processes, and can be simply extended to wafer-scale device manufacturing. Ultimately, we present a generalizable method that opens a route to bottom-up integration of nanomaterials for industry-scale integrated circuits.

## Methods

### Device fabrication

We implemented and tested the graphene-based placement method on (1) SiC and on (2) highly insulating Si with an SiO_2_ capping layer having a thickness of 300 nm. For (2), see the implementation example in Fig. 5 where we manufactured the SiO_2_/Si substrates with local bottom gate electrodes. To that end, we first patterned a poly methyl methacrylate (PMMA) layer by e-beam lithography, followed by SiO_2_ etching, metal deposition, and lift-off in hot acetone. In the next step, we deposited 30 nm of Al_2_O_3_ at 250 °C by using atomic layer deposition. We then transferred chemical vapor deposition (CVD)-grown graphene^25^ by a PMMA-assisted technique^15^ onto the gate stack. Starting the transfer by first spin coating PMMA (A4, 950 PMMA, MicroChem Corp.) on pre-cut pieces of CVD graphene on a copper foil (Graphene Laboratories, Inc.), we then placed the graphene on copper foil into copper etchant (PC Copper Etchant-200, Transene Company, Inc.) for 20 min. After copper was dissolved, we transferred the floating PMMA/graphene bilayer into two subsequent water baths (high purity, de-ionized water, with a resistivity of 18 MΩ-cm) to remove residual chemicals. The cleaned PMMA/graphene was then scooped onto the target substrate, blown dry with clean nitrogen, and baked at 100 °C for several hours to remove residual water. We then defined graphene contact areas by patterning a negative tone resist bilayer (PMMA/HSQ), followed by an oxygen plasma step to etch exposed graphene areas. Subsequently, we defined large metallic contact pads by e-beam lithography. This step was followed by metal thin film evaporation (5 nm Ti/50 nm Au), and a lift-off step. The larger contact pads were located on the edges of the sample to function as interfaces for the external measurement system. We then applied an alternating voltage (0.1–10 MHz, 1–10 V_p–p_) to the graphene contacts by means of a waveform generator (DS345, Stanford Research Systems) via co-planar microwave probes (GGB Industries, Inc.) operated in a ground-source-ground configuration. In order to avoid material deposition due to fringing fields away from the intended deposition locations, the amplitude of the deposition voltage was chosen such that we used the minimum value at which assembly actually occurs. Upon application of the voltage, we deposited about 20 µl of highly diluted nanomaterial solution onto the sample surface for periods of 1–10 mins. The deposition process was completed by rinsing the sample sequentially with water and isopropyl alcohol. Following nanomaterial deposition, we defined a mask on top of the as-deposited material by patterning a negative tone resist bilayer (PMMA/HSQ), followed by an oxygen plasma step to etch the exposed graphene areas. In a final step, we patterned metallic contacts to the deposited nanomaterial, followed by metal evaporation (5 nm Ti/50 nm Au) and a lift-off step, for enabling electrical characterization with the external measurement setup.

### Nanomaterial dispersion preparation

The semiconducting CNT solution was prepared as previously reported via density gradient ultracentrifugation, with >99% semiconducting purity and refined average diameter of 1.6 nm^26–31^. MoS_2_ dispersion preparation began with 60 mg of MoS_2_ powder (American Elements, Inc.) and 15 mL ethanol-water co-solvent (10 mL ethanol and 5 mL de-ionized water) being placed in a 50 mL plastic conical tube with a sealed setup^32^ to avoid solvent evaporation and exfoliated by ultrasonication (Model 500 Sonic Dismembrator, Fisher Scientific) at ~30 W for 1 h in an iced bath. The resulting MoS_2_ dispersions were then centrifuged at 5000 rpm for 30 min to remove unexfoliated MoS_2_ powder (Avanti J-26 XP, Beckman Coulter, Inc.) and the supernatant was carefully decanted. CdSeS/ZnS alloyed quantum dots in aqueous solution (COOH functionalized, fluorescence wavelength maximum *λ*_em_ = 575 nm, 6 nm diameter, 1 mg/ml in H_2_O) were purchased from Sigma-Aldrich, Inc.

### Material and device characterization

In order to identify deposited nanomaterials in situ, we have acquired hyperspectral images of as-fabricated samples by combining Raman and photoluminescence micro-spectroscopies with a confocal laser scanning microscope (alpha300 RAS, WITec GmbH). In addition, we have performed in the same measurement system topographical characterization of the samples by means of atomic force microscopy. Scanning electron microscopy was used to image samples at each step of device fabrication. As-acquired electron scanning micrographs have been color-coded to indicate image constituents (substrate, electrodes, materials, etc.), without any further processing or contrast enhancement. Electrical transport measurements have been performed under N_2_ flow by using a probe station (FWP6, LakeShore Cryotronics, Inc.) equipped with tungsten probes having a 50 μm tip radius mounted on ceramic blade probe bodies, and a semiconductor parameter analyzer (B1500A Semiconductor Device Analyzer, Agilent Technologies, Inc.).

### Electric field simulation

We perform quasi-electrostatic field simulation using COMSOL Multiphysics (COMSOL, Inc.) to evaluate the dielectrophoretic force field. We set up the simulation domain identical to the experimental conditions (physical dimensions, frequencies, applied voltages) using literature values for all relevant physical quantities (electrical conductivity, relative permittivity). Modeling the polarized bound charges of a rod-shaped particle immersed in an electric field as an induced dipole, we calculate the time-averaged dielectrophoretic force acting upon a CNT from the simulated electric field by the following relation^33^:

$${\mathop{F}\limits^{\rightharpoonup}}_{\mathrm{DEP}} = \frac{{\pi d^2l}}{8}\varepsilon _{\mathrm{m}}{\mathrm{Re}}\left[ {\mathrm{CM}} \right]\nabla \left| {{\mathop{E}\limits^{\rightharpoonup}}_{\mathrm{rms}}} \right|^2,$$

In this expression, *d* and *l* denote the diameter and length of the tube, respectively, CM represents the Clausius–Mossotti factor given as:

$${\mathrm{CM}} = \frac{{\varepsilon _{\mathrm{t}} - \varepsilon _{\mathrm{m}}}}{{\varepsilon _{\mathrm{m}} + \left( {\varepsilon _{\mathrm{t}} - \varepsilon _{\mathrm{m}}} \right)L}}$$

and *ε*_t_ and *ε*_m_ are the complex permittivity of the CNT and surrounding medium, respectively. The complex permittivity is given by $\varepsilon = \varepsilon _0\varepsilon _{\mathrm{r}} - {\mathrm{i}}\sigma /\omega$, where *ε*_r_ denotes the relative permittivity of the material, *ε*_0_ the free space permittivity (8.85 × 10^−12^ F/m), *σ* (S/m) is the conductivity, and *ω* (rad/s) the angular frequency. Our simulations assume a depolarization factor *L* equal to 10^−5^, a tube diameter *d* equal to 1 nm and a length *l* equal to 500 nm^34^. Under those conditions, all metallic and most semiconductor CNTs experience positive DEP (Re(CM) > 0), that is, the nanomaterial is attracted to electric field intensity maxima at the edges of the electric contact. For simplicity, a Clausius–Mossotti factor of 1 was used in the simulations.

## Electronic supplementary material

Supplementary Information

## Data Availability

The datasets generated during and analyzed during the current study are available from the corresponding author on reasonable request.
