# Supplementary material for: Graphene-enabled and directed nanomaterial placement from solution for large-scale device integration
Source: Nat Commun. 2018 Oct 5;9:4095. doi: 10.1038/s41467-018-06604-4 (PMC6173726; doi:10.1038/s41467-018-06604-4)
Supplement: Supplementary file 1 — Supplementary Information [file 41467_2018_6604_MOESM1_ESM.docx]

Supplementary Information

Graphene-enabled and directed nanomaterial placement from solution for large-scale device integration

Michael Engel^1^, Damon B. Farmer^2^, Jaione Tirapu Azpiroz^1^, Jung-Woo T. Seo^3^, Joohoon Kang^3^, Phaedon Avouris^2^, Mark C. Hersam^3^, Ralph Krupke^4,5,6^, Mathias Steiner^1,2*^

^1^ IBM Research, Rio de Janeiro, RJ 22290-240, Brazil

^2^ IBM Research, Yorktown Heights, NY 10598, USA

^3^ Department of Materials Science and Engineering and Department of Chemistry, Northwestern University, Evanston, IL 60208, USA

^4^ Institute of Nanotechnology, Karlsruhe Institute of Technology, 76021 Karlsruhe, Germany

^5^ DFG Center for Functional Nanostructures (CFN), 76028 Karlsruhe, Germany

^6^ Institut für Materialwissenschaft, Technische Universität Darmstadt, 64287 Darmstadt, Germany

[^*^msteine@us.ibm.com](mailto:*msteine@us.ibm.com), [mathiast@br.ibm.com](mailto:mathiast@br.ibm.com)


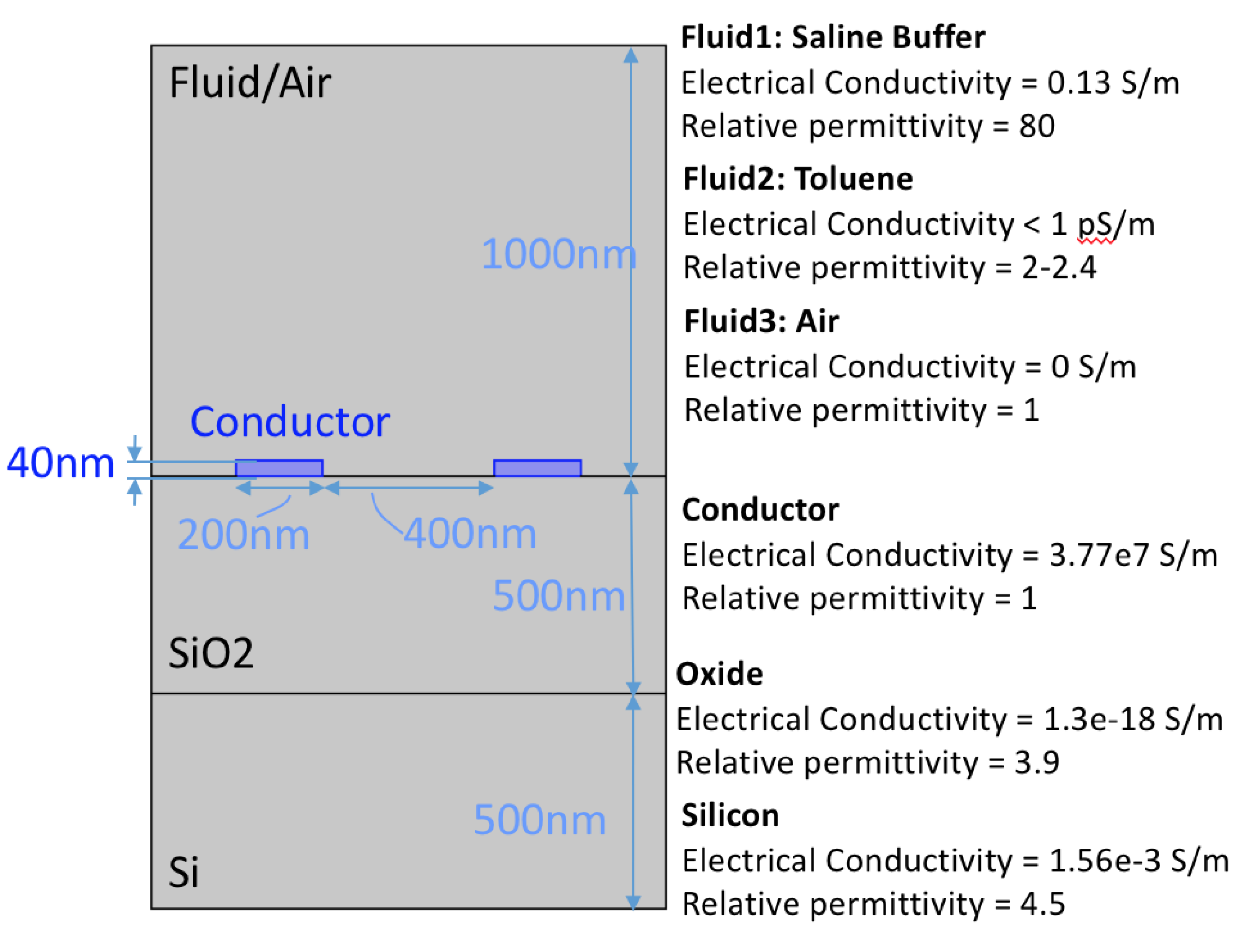


Supplementary Figure 1 | Layout for electric field simulation. Material stack used for quasi-electrostatic field simulation discussed in the main manuscript, including relevant physical parameters and dimensions.


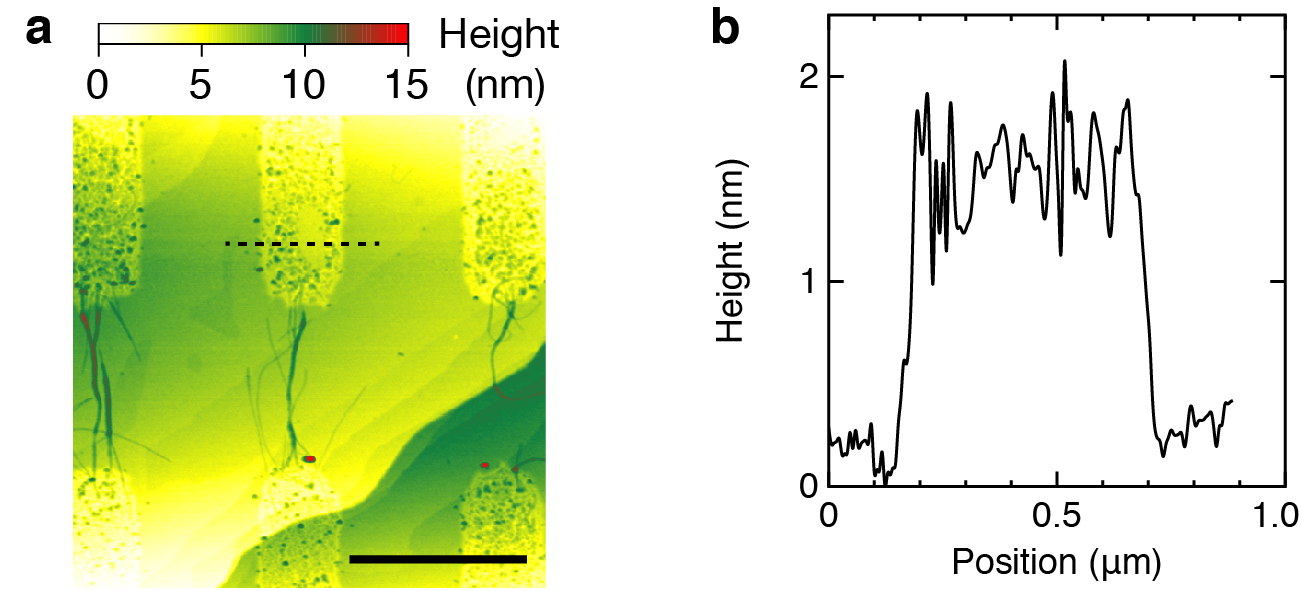


Supplementary Figure 2 | Topographical analysis of graphene electrode thickness. a AFM image taken after CNT deposition. The dashed line in the image indicates the position where the cross section was taken. Length of scale bar: 1µm. b AFM cross section taken at the position indicated by dashed line in a. The cross section reveals a graphene thickness of about 1.5nm which matches the values obtained from cross sections taken at other locations.


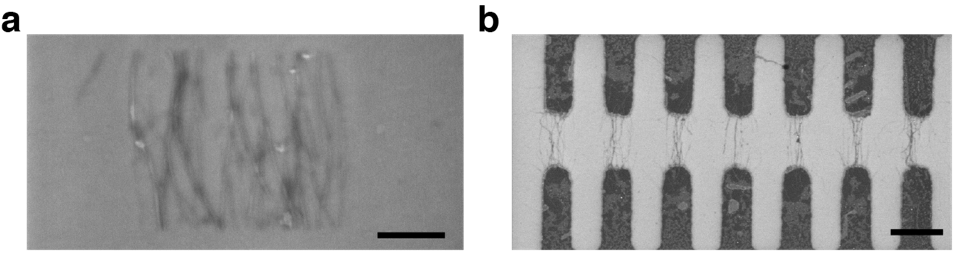


Supplementary Figure 3 | High-resolution SEM images of sub-micrometer scale CNT assembly. a Scanning electron micrograph taken after removal of graphene placement electrodes allows for estimating CNT density. Scale bar: 200nm. b Scanning electron micrograph of sub-micron CNT assembly using graphene placement electrodes. Scale bar: 1µm.


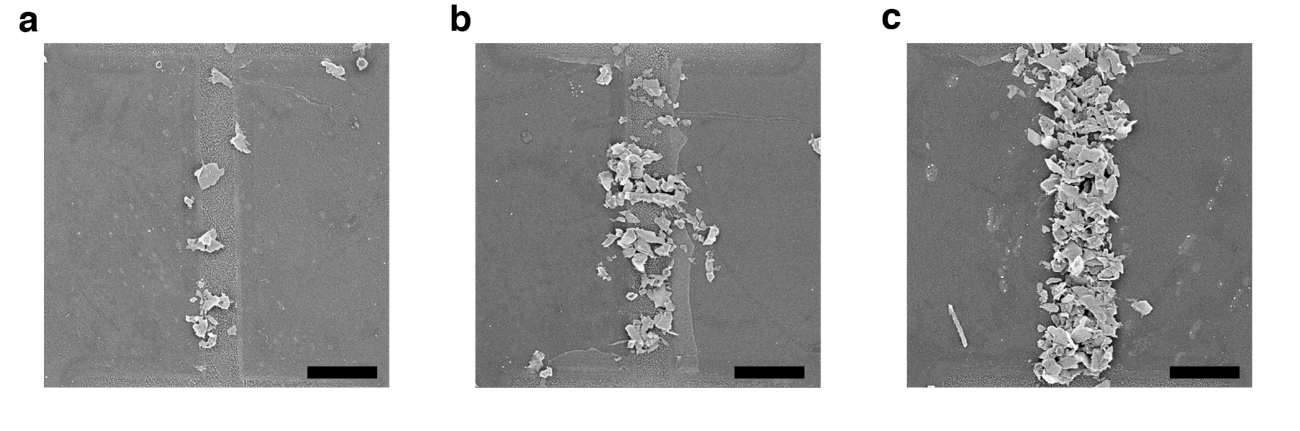


Supplementary Figure 4 | Assembled material density as function of deposition voltage. Scanning electron micrographs of as-deposited MoS_2_ flakes after a low-density deposition (*f*=1MHz, *V*_pp_=1V, *t*=5min), b medium-density deposition (*f*=1MHz, *V*_pp_=5V, *t*=5min), and c high-density deposition (*f*=1MHz, *V*_pp_=10V, *t*=5min). Length of scale bars: 1µm.


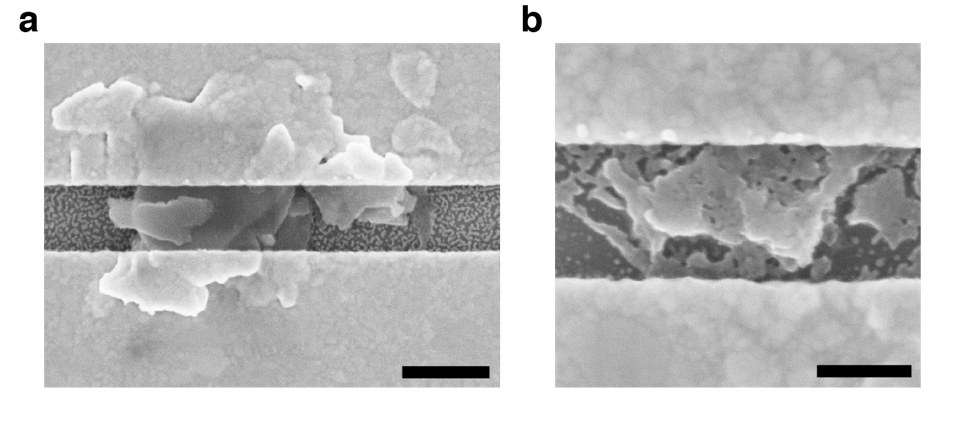


Supplementary Figure 5 | Fully processed devices made of 2D and 0D material. a Scanning electron micrograph of a MoS_2_ device contacted by metallic electrodes after graphene electrode removal. Length of scale bars: 400nm. b Scanning electron micrograph of a quantum dot device contacted by metallic electrodes after graphene removal. Deposition parameters for all devices are: *f*=1MHz, *V*_pp_=5V, and *t*=5min. Length of scale bars: 200nm.


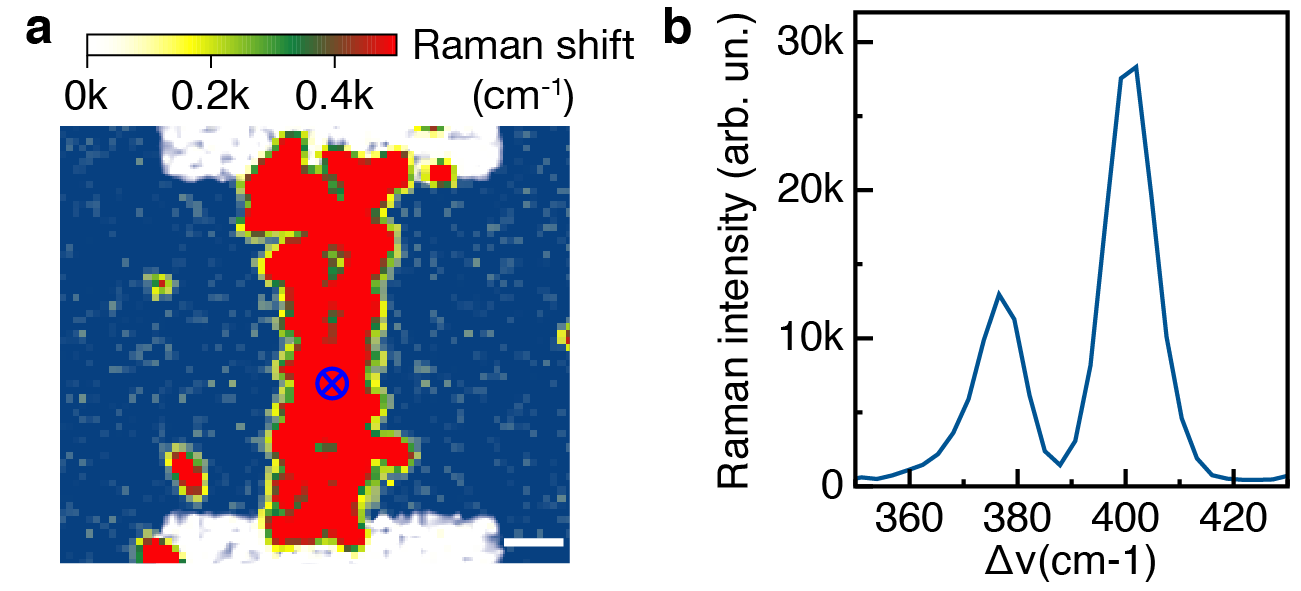


Supplementary Figure 6 | Raman mapping and spectroscopy of assembled MoS_2_. a Raman 2D intensity false color image (white: 20 arb. units, blue: 100 arb. units) indicating graphene areas, overlaid by a Raman intensity false color image spectrally integrated at (375±10)cm^-1^ indicating position of few-layer molybdenum disulfide. Scale bar: 1µm. b Raman spectrum taken at the location marked in a. The spectrum exhibits the E^1^_2g_ and A_1g_ Raman active modes in MoS_2_^1^.


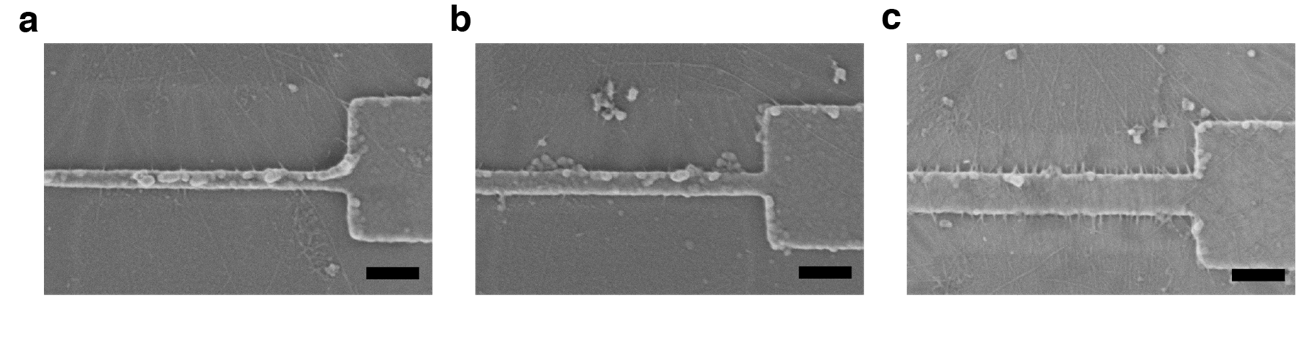


Supplementary Figure 7 | Scaled CNT FETs with local bottom gates. Scanning electron micrographs of carbon nanotubes deposited across local bottom gates having lengths of a 40nm, b 67nm, and c 130nm, respectively. Deposition parameters for all devices are: *f*=1MHz, *V*_pp_=3V, and *t*=5min. Length of scale bars: 200nm.


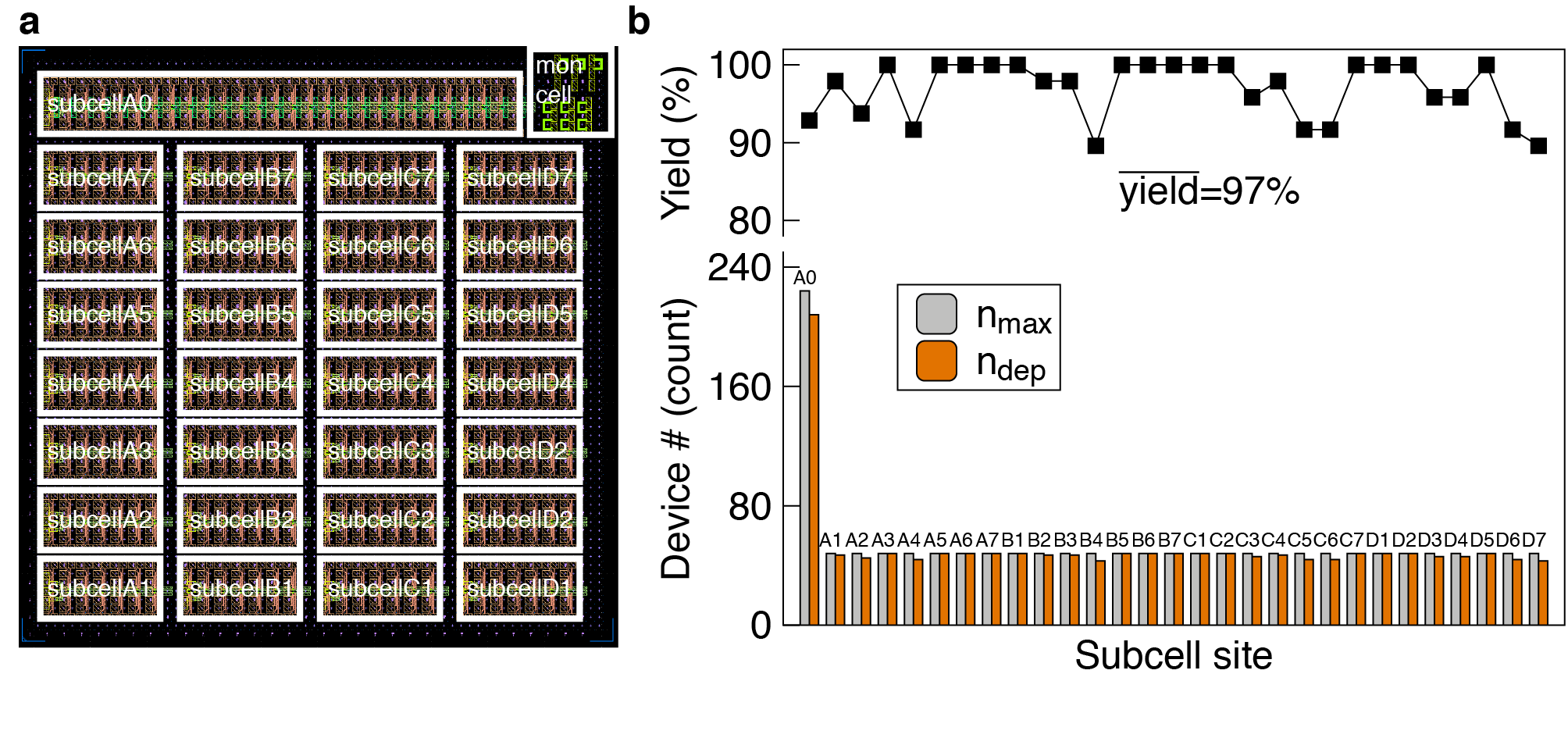


Supplementary Figure 8 | Deposition yield analysis at large scale. a CAD layout of a wafer die containing 25 subcells and 1 monitor cell featuring a total of 1568 deposition sites. b Deposition yield analysis of a representative die reveals an average yield of 97% across the entire die.

**References**

1. Li, H. *et al.* From Bulk to Monolayer MoS2: Evolution of Raman Scattering. *Adv Funct Mater* **22,** 1385–1390 (2012).
